# Exploration of Major Cognitive Deficits in Medication-Free Patients With Major Depressive Disorder

**DOI:** 10.3389/fpsyt.2019.00836

**Published:** 2019-11-12

**Authors:** Jin Liu, Qiangli Dong, Xiaowen Lu, Jinrong Sun, Liang Zhang, Mi Wang, Ping Wan, Hua Guo, Futao Zhao, Yumeng Ju, Danfeng Yan, Haolun Li, Han Fang, Weilong Guo, Mei Liao, Xiangyang Zhang, Yan Zhang, Bangshan Liu, Lingjiang Li

**Affiliations:** ^1^Department of Psychiatry, The Second Xiangya Hospital, Central South University, Changsha, China; ^2^Mental Health Institute of Central South University, China National Clinical Research Center on Mental Disorders (Xiangya), China National Technology Institute on Mental Disorders, Hunan Technology Institute of Psychiatry, Hunan Key Laboratory of Psychiatry and Mental Health, Changsha, China; ^3^Department of Psychiatry, Zhumadian Psychiatric Hospital, Zhumadian, China; ^4^CAS Key Laboratory of Mental Health, Institute of Psychology, Chinese Academy of Sciences, Beijing, China

**Keywords:** depression, cognitive function, neurocognition, executive function, processing speed

## Abstract

**Background:** Major depressive disorder (MDD) is associated with a wide range of cognitive deficits. However, it remains unclear whether there will be a major cognitive deficit independently caused by depression at acute episodes of MDD.

**Method:** A comprehensive neurocognitive test battery was used to assess the executive function, processing speed, attention, and memory in 162 MDD patients and 142 healthy controls (HCs). A multivariate analysis of variance, hierarchical regression analyses and general linear regression analyses were used to explore the possible major cognitive deficits and their predictor variables.

**Results:** MDD patients showed extensive impairment in all four cognitive domains. Impairment of executive function and processing speed were found to persist even with other cognitive domains and clinical variables being accounted for. Executive function and processing speed were further predicted by total disease duration and depression severity, respectively.

**Conclusions:** Executive function and processing speed may be two distinct major deficits at acute episodes of MDD. Furthermore, the executive function is likely originated from the cumulative effect of disease duration and processing speed is possibly derived from the temporary effect of current depressive episode.

## Introduction

Cognitive impairment is one of the most common symptoms of major depressive disorder (MDD) (1), and approximately 90% of MDD patients complain about cognitive problems (2). Previous studies have established a wide range of cognitive impairment features in MDD, involving four major cognitive domains: attention, memory, executive function, and processing speed (3–5). These deficits have been repeatedly reported to be associated with poor prognosis of MDD, including decreased social functioning and remission rate, increased risk of relapse, and increased disease burden (6, 7). However, although many studies have investigated the characteristics of cognitive function in MDD, little is known about the inter-relationship between these cognitive deficits. Specifically, it is still unclear whether the deficits in different cognitive domains are directly caused by depression or mainly originated from impairments in one or more basic domains, that is, some major cognitive deficits.

Major cognitive deficits are deficits directly caused by the disease. They are independent of other deficits and may lead to some secondary deficits in other cognitive domains (8, 9). The identification of major cognitive deficits in MDD is not only helpful for the differential diagnosis of MDD from other psychiatric disorders, such as schizophrenia and bipolar disorder, but also helpful for establishing the cognitive targets for antidepressant treatment. However, many previous studies on the cognitive function of MDD have assessed only one or several cognitive domains, resulting in insufficient information to identify major deficits. Only a limited number of studies have conducted a comprehensive assessment of the cognitive domains and investigated the major deficits in MDD (8–12). Most of these studies focused on late-life depression (LLD). Two studies reported processing speed as the most promising major deficit in LLD, with executive function as the second most (10, 11), as it mediates most of the relationships between clinical variables and other cognitive domains. However, evidence from the mediating effect between clinical variables and other cognitive domains is indirect evidence supporting major deficits. To confirm the role of major deficits, it is necessary to directly test the independent contribution of MDD to a particular cognitive domain with other cognitive domains being controlled. Two other studies conducted this analysis and found that processing speed and executive function accounted for a large proportion of depression-related variance in other cognitive domains, such as episodic memory, visuospatial memory, and language skills (8, 12). These results provided direct support for taking processing speed and executive function as major deficits in LLD.

As far as we know, the only study that focused on MDD in younger adults (18–60 years) (9) identified attention as the major deficit. Although the authors explained that most of the attention tests used in their study were related to processing speed, the mediating effect of attention on depression-related executive function did show potential differences in MDD-related cognitive deficits between younger (<60 years) and elder (>60 years) adults (9). Considering the relatively small sample size (about 30 in either group) and the lack of differentiation between attention and processing speed in this study, further studies are needed to recruit a larger sample and use tests that evaluate all four major cognitive domains.

Another key aspect of cognitive function in MDD is the impact of clinical variables on different cognitive domains. The relationship between clinical variables and cognitive domains not only indicates the origins of cognitive deficits (9) but also guides potential interventions (13). For example, a deficit that is mainly predicted by the severity of depression may be due to the state-dependent effects of current depressive episode (1, 14, 15), while a deficit predicted by disease duration or episodes may result from the cumulative detrimental effects of previous depressive episodes (16–19). The former deficit might be improved only by treating current depression, while the latter deficit may require additional interventions, such as domain-specific cognition training (20).

To explore the major cognitive deficits, we carried out this study using a comprehensive battery of neurocognitive tests in large adult medication-free MDD and matched healthy control (HC) samples. The goals of our study were to assess the major cognitive deficits independently caused by MDD and their predictor variables. As noted above, most existing studies reported processing speed and executive function as major deficits of MDD. Thus, we hypothesized that both processing speed and executive function would be the major cognitive deficits of MDD and they would derive from different disease origins.

## Methods

### Participants

One hundred and sixty-two patients with MDD and 142 HCs were recruited from the Zhumadian Psychiatric Hospital (Henan, China) and its nearby communities from 2013 to 2018. The common inclusion criteria for the two groups were: 1) aged 18 to 55 years; 2) education ≥6 years; 3) right-handed. For the MDD group, additional inclusion criteria were: 1) diagnosed with MDD by an attending psychiatrist using Structured Clinical Interview for DSM-IV (SCID-IV); 2) currently had at least moderate depression severity, expressed by a 24-item Hamilton Depression Rating Scale (HAM-D_24_) score ≥20; 3) no psychotropic medication for at least 2 weeks (6 weeks for fluoxetine) before enrollment.

Additional inclusion criteria for HCs were: 1) no current or lifetime diagnosis of any psychiatric disorders or substance abuse; 2) HAMD_24_ ≤ 7. The exclusion criteria for the two groups were: 1) any other DSM-IV psychiatric disorder; 2) pregnancy or breastfeeding; 3) history of head injury or neurological disorder; 4) DSM-IV alcohol/drug dependence; 5) color-blindness; 6) and had received any similar neurocognitive assessments in the past 12 months.

This study was approved by the Medical Ethics Committees of the Second Xiangya Hospital of Central South University and the Zhumadian Psychiatric Hospital. All participants provided written informed consent.

### Neurocognitive Assessment

A comprehensive battery of neurocognitive tests was administrated to all participants. Based on previous literature, we divided the subtests of these tests into four cognitive domains: executive function, attention, processing speed, and memory (11, 21, 22). The composite score for each domain was calculated based on the summation of the standardized Z-score of the corresponding subtests.

*Executive function.* This domain included six subtests: the digit span backward (DSB) test from the Wechsler Adult Intelligence Scale-Revised (WAIS-R) (correctly completed number of trails), the color-word interference condition of the Stroop test (total correct number), the categories completed and perseverative errors from the Wisconsin Card Sorting Test (WCST) (total correct number), the Part B of the Trail-Making Test (TMT-B) (time to completion), and the Semantic Verbal Fluency (animals) test (total correct number) (23). These subtests cover three core subcomponents of executive function: working memory, inhibition control, and cognitive flexibility (24). Specifically, DSB assesses working memory; the color-word interference condition of the Stroop test assesses inhibition control; the categories completed and perseverative errors from the WCST, the TMT-B, and the Semantic Verbal Fluency (Animals) test assess cognitive flexibility (24, 25).

*Attention.* This domain included two subtests: the word condition of the Stroop test (total correct number) and the digit span forward (DSF) test from the WAIS-R (correctly completed number of trails). The two subtests mainly assess sustained attention.

*Processing speed.* This domain included two subtests: Part A of the Trail-Making Test (TMT-A) (time to completion) and the color condition of the Stroop test (total correct number).

*Memory.* This domain included three subtests: the Visual Memory Test (total correct number), the Intelligent Memory Test (total correct number), and the Associative Memory Test (total correct number) of the Wechsler memory scale. The three subtests evaluated three subcomponents of memory: visual memory, intelligent memory, and associative memory (26).

### Statistical Analysis

We summed the standardized Z-scores of different subtests to generate a standardized score for each cognitive domain. The Z-scores were calculated using the following equation:

$$Z=\frac{X_{\mathrm{individual}}-{\bar{X}}_{\mathrm{controls}}}{\sigma_{\mathrm{controls}}}$$

X_individual_ represents the raw score of each individual, ${\bar{X}}_{\mathrm{controls}}$ represents the average value of HCs, and σ_controls_ represents the standard deviation of HCs. To ensure that higher Z-score represents better performance in all subtests, we reversed the values of the subtests, in which lower raw values (TMT-A and perseverative errors from the WCST) indicate better performance. Importantly, in this study, the average z-score composite was applied to perform different numbers of subtests for each domain. The internal consistency of different subtests in each cognitive domain was assessed by Cronbach’s α. The normality of the variables was tested by the Kolmogorov-Smirnov test. For skewed variables, log or square root transformations were applied to achieve normality.

The relationships between cognitive performances were assessed by Pearson correlation analyses. A multivariate analysis of covariance (age, gender, and education) (MANCOVA) was conducted to investigate the difference between MDD and HC group in all four cognitive domains.

Furthermore, a series of hierarchical regression analyses were conducted to investigate whether there were major cognitive deficits. In each hierarchical regression model, one cognitive domain was selected as the dependent variable, and the demographic variables, the other three cognitive domains, the group membership (MDD vs. HCs) were entered into the model one by one as independent variables in three steps.

Multiple regression analyses were conducted to assess the effects of predictor variables on each cognitive domain. The statistical significance level was set to p < 0.05 (two-tailed). All statistical analyses were conducted in SPSS 24.0.

## Results

### Demographic and Clinical Characteristics of the Participants

The demographic and clinical characteristics of MDD and HC groups are shown in **Table 1**. There were no significant differences in age, gender, and education between the MDD and HC groups. Participants in the MDD group were moderately-to-severely depressed.

**Table 1:** Demographic and clinical characteristics of MDD and HC groups.

Variable	Patients (n = 162) mean (SD)	Controls (n = 142) mean (SD)	T or χ²	P
**Demographic characteristics**				
Age (years)	35.1 (9.55)	34.86 (8.78)	T = 0.23	.816
Male n (%)	72 (44.4)	64 (45.1)	χ² = 0.01	.913
Female n (%)	90 (55.6)	78 (54.9)		
Education (years)	10.45 (3.41)	11.01 (3.47)	T = −1.41	.160
**Mood ratings**				
HAM-D	31.56 (7.28)	1.39 (1.76)	T = 48.09	**<.001**
HAM-A	18.33 (6.31)	1.08 (1.58)	T = 39.80	**<.001**
**Clinical characteristics**				
Age of onset (years)	31.94 (9.71)	–		
Number of episodes (n)	2.15 (1.51)	–		
Single n (%)	64 (39.5)	–		
Recurrent n (%)	98 (60.5)	–		
Total illness duration (months)	40.36 (50.64)	–		

Bold values indicate statistical significance.

HC,healthy control; MDD,major depressive disorder.

### Characteristics of Neurocognitive Function in MDD

The Cronbach’s alphas of the four cognitive domains ranged from 0.655 to 0.778, indicating a relatively high internal consistency between these measures (**Table 2**). MANCOVA analysis revealed a significant overall difference in cognitive function between the two groups (F_(4,__296)_ = 43.79, P < 0.001, Partialη^2^^=^ 0.372, **Table 2**). Post hoc analysis demonstrated that MDD patients performed significantly worse than HCs in all four cognitive domains (all P < 0.001).

**Table 2:** Comparisons between MDD and HC groups in the four cognitive domains (Z-score) (MANCOVA test).

Cognitive domain	Depressed (n = 162) mean (SD)	Control (n = 142) mean (SD)	F	P	Partial η^2^	Cronbach’s α
Executive function	−0.49 (0.62)	0.00 (0.64)	63.93	**<.001**	.461	.732
Attention	−0.58 (0.93)	0.00 (0.84)	44.84	**<.001**	.375	.655
Processing speed	−0.77 (0.98)	0.00 (0.82)	28.88	**<.001**	.279	.778
Memory	−0.81 (0.22)	0.00 (0.18)	47.46	**<.001**	.388	.763

Bold values indicate statistical significance.

HC,healthy control; MDD,major depressive disorder.

### Identification of the Major Cognitive Deficits in MDD

Performances in the four cognitive domains were highly inter-correlated with each other (**Table 3**). We developed a series of hierarchical regression models to assess the independent contributions of group membership to each cognitive domain. If the group membership continues to make a significant contribution to the variance of the dependent variable even after accounting for the demographic variables and the other three cognitive domains, then this dependent variable is considered as a major cognitive deficit. The results showed that, even when other cognitive domains and clinical variables were accounted for, group membership continued to explain a significant proportion of the variance in executive function (P < 0.001) and processing speed (P < 0.001) (**Table 4**).

**Table 3:** Correlations among the four cognitive domains in the whole sample (MDD+HC).

	Executive function	Attention	Processing speed	Memory
Executive function	–	.652**	.540**	.657**
Attention	–	–	.583**	.599**
Processing speed	–	–	–	.540**
Memory	–	–	–	–

**P < 0.01.

HC,healthy control; MDD,major depressive disorder.

**Table 4:** Results of hierarchical regression analyses examining different cognitive performances in depressed and control sample when accounting for other cognitive domains and demographic variables.

Dependent variable	Independent variables	R^2^	R^2^ change	F for R^2^ change	P
Executive function	Age, gender, education	.352	.352	54.24	**<.001**
	+Attention, Processing speed, Memory	.581	.229	54.06	**<.001**
	+Group: patient vs. control	.600	.020	14.67	**<.001**
Processing speed	Age, gender, education	.142	.141	16.61	**<.001**
	+Executive function, Attention, Memory	.420	.278	47.49	**<.001**
	+Group: patient vs. control	.446	.025	13.54	**<.001**
Attention	Age, gender, education	.296	.296	42.08	**<.001**
	+Executive function, Processing speed, Memory	.551	.255	46.25	**<.001**
	+Group: patient vs. control	.553	.002	1.16	.296
Memory	Age, gender, education	.323	.323	47.70	**<.001**
	+Executive function, Attention, Processing speed	.529	.215	46.21	**<.001**
	+Group: patient vs. control	.541	.002	1.61	.206

Bold values indicate statistical significance.

### Identification of the Independent Predictor Variables of Major Cognitive Domains in MDD

As a previous study has demonstrated that the effect of age of onset on cognitive function is mediated by age (11), we conducted a series of hierarchical regressions to investigate the mediating effect of age on the contribution of age of onset to the two major cognitive domains. We found that age of onset failed to explain any significant proportion of the variance in any of the two major cognitive domains when age was accounted for (all P > 0.05), while age continued to explain the variance in executive function when age of onset was accounted for (**Table S1**). Thus, we took age as an independent risk factor and deleted age of onset in the subsequent analyses.

We developed two regression models using age, gender, education, HAM-D, HAM-A, total illness duration and number of episodes to predict cognitive performances. The results showed that both age and education made significant independent contributions to executive function (both P < 0.001) and processing speed (both P = 0.008), while total disease duration only made independent contribution to executive function (P = 0.028) and the HAM-D only made independent contribution to processing speed (P = 0.037) (**Table 5**).

**Table 5:** Results of regression analyses determining the effect of predictor variables on executive function and processing speed.

	Variables	β	t	P	R^2^	F	P
Executive function	Age	−.271	−3.99	**<.001**	.387	13.77	**<.001**
	Gender	.065	.98	.326			
	Education	.435	6.44	**<.001**			
	HAM-D	−.101	−1.16	.249			
	HAM-A	−.009	−.098	.922			
	Total illness duration	−.232	−2.22	**.028**			
	Number of episodes	.094	.89	.373			
Processing speed	Age	−.215	−2.70	**.008**	.164	4.30	**<.001**
	Gender	.103	1.33	.184			
	Education	.213	2.70	**.008**			
	HAM-D	−.215	−2.10	**.037**			
	HAM-A	.028	.27	.791			
	Total illness duration	.079	.64	.521			
	Number of episodes	−.045	−.37	.711			

Bold values indicate statistical significance.

## Discussion

This study utilized a comprehensive cognitive assessment battery to investigate the characteristics and influencing factors of cognitive function in MDD. Our results are interesting in the following areas. First, widespread cognitive impairment was detected in all four cognitive domains of adult MDD patients. Among them, executive function and processing speed are the domains independently predicted by group membership after accounting for the other three cognitive domains, suggesting that these two domains may be the major cognitive deficits in MDD. Second, among the four cognitive domains, executive function and processing speed are the only two domains independently predicted by disease-related variables. Specifically, total disease duration predicted executive function and depression severity predicted processing speed. These results indicate that the major deficits in executive function and processing speed may be derived from different origins, one from the cumulative detrimental effect of disease duration, and the other from the state-dependent influence of current depressive episode. Third, age and education are the only two predictors for all the two major cognitive deficits in MDD, implying a strong effect of demographic variables on cognitive functions in MDD.

The results of executive function and processing speed as major deficits in MDD are consistent with previous studies investigating major deficits in LLD (8, 10–12). Importantly, our study differs from previous studies in that it provided dual evidence for confirming the major deficits: independent prediction from MDD membership (direct evidence) and independent prediction from MDD-related variables (indirect evidence). The two pieces of evidence were mutually confirmed and provided robust support for taking executive function and processing speed as the major deficits. Interestingly, we were unable to further clarify which one of the two major deficits is more basic, as neither of them could be fully accounted for by the other three cognitive domains. This might be explained by the diverse origins of these two deficits. As discussed above, deficit in executive function is likely linked to the cumulative toxic effect of long-term disease on the brain, while the deficit in processing speed is likely associated with the temporary effect of current depressive episode.

Executive function is a higher-level cognitive process that regulates and integrates other cognitive processes to guide goal-directed behaviors (23). Executive function is usually considered as a function of the prefrontal cortex and sometimes referred to as “frontal lobe task”, although other brain regions and networks are also involved (24, 27, 28). Previous studies have reported toxic effects of depressive episodes on the brain, including increased oxidative stress and neural inflammation (29, 30), and decreased neural plasticity (31), resulting in structural and connective alterations in PFC and other brain regions (32–34). These changes in PFC are often associated with blunted performance on executive function tests (35). Even after the clinical remission, toxic effects persist and may accumulate with future depressive episodes (36), resulting in enduring impaired executive function in depression remission (37–40) and progressively deteriorated executive function in recurrent depressive episodes (41). Consistent with these reports, our results also revealed a strong correlation between total disease duration and executive function, and longer disease duration predicted worse executive function, suggesting a cumulative detrimental effect of disease duration on executive function.

In contrast with the enduring and cumulative toxic effects of depression on the prefrontal cortex and executive function, the effect of depression on processing speed seems to be state-dependent. The correlation between depression severity and processing speed was in line with the clinical picture of slowed thinking in acute depression (42). With effective treatment, the slowed thinking is typically significantly improved in remitted MDD patients (42, 43), consistent with the evidence of improvement in processing speed with antidepressant treatment (44, 45) or psychological training. A possible explanation for the provisionality of the effect of depression on processing speed may lie in the motor speed hypothesis. It has been proposed that decreased processing speed in depression may be attributed to the psychomotor retardation, a common symptom of depression, affects both emotional and cognitive processing (46, 47). Moreover, the effect of current depression severity on processing speed was further supported by the mediating effect of processing speed on the relationships between depression severity and other cognitive domains (48). Taken together, the deficit in processing speed is likely derived from the reversible effects of current depressive episodes. Previous neuroimaging studies also identified slowed processing speed as a major cognitive deficit in LLD with certain fronto-striatal disconnection and prefrontal white matter hyperintensities (49, 50). However, studies addressing the functional or structural brain substrates of slowed processing speed in middle-aged MDD patients are still lacking.

Another important finding of this study is the independent contributions of age and education to these two major cognitive deficits. Specifically, age and education are the strongest predictors for these two major cognitive deficits in MDD. In addition, age mediated the effect of age of onset on all the four cognitive domains. These results, consistent with the findings in LLD studies (10, 11), suggest strong effects of demographic variables on cognitive function in MDD, calling for attention to the demographic diversity when assessing the cognitive function in MDD in the future.

Unexpectedly, we did not find an independent contribution of gender, comorbid anxiety, and number of episodes to the two major cognitive deficits in depression. For gender, only limited studies have addressed gender differences in cognitive performance. Although different performances in psychomotor speed, verbal learning, memory, visuoconstruction, and visual perception have been reported in healthy women and men, both studies in healthy samples and LLD samples found no significant difference in executive function (51, 52). Our result was in line with the previous study of LLD (51). Nevertheless, large sample studies are needed to clarify the gender-related cognitive differences. For comorbid anxiety, one study aimed to evaluate the contributions of anxiety symptoms on cognitive performances in MDD patients revealed that there are no significant differences in all neurocognitive tests between MDD patients with and without comorbid anxiety (53). Another study reported that anxiety symptoms fail to predict objective cognitive performance in MDD patients (54). These shreds of evidence implied that anxiety symptoms might virtually contributed insufficient on cognitive performance in MDD. Generally, number of episodes is positively correlated with total illness duration, with more episodes accompanying longer illness duration. Surprisingly, we found an independent contribution of total illness duration to executive function, while no contribution of number of episodes to these two major cognitive deficits. We believe this might be associated with the fact that total illness duration provides more detailed information as compared with number of episodes. For instance, although parts of MDD patients are classified as “first-episode”, their total illness duration of varies significantly.

The present study is strengthened by evaluating cognitive performance in a relatively large sample of medication-free adult MDD patients and HCs. Definitively, antidepressants would have varying effects across different cognitive domains and depression. Therefore, to reveal the nature of cognitive deficit in both acute and medication-free MDD is of importance. Moreover, only one study examining major cognitive deficits in smaller younger samples followed the previous studies focusing on LLD (9). The present study provided further evidence to identify the potential major cognitive deficits in a relatively large adult MDD sample.

An important limitation of this study is the potential overlap between different cognitive functions. For instance, subtests that evaluate executive function (e.g., TMT-B, DSB, Stroop color-word interference, among others) may also involve other cognitive functions, such as processing speed and attention. This may lead to potentially exaggerated correlations between these cognitive domains and the difficulty in detecting hierarchical relationships between them. Notwithstanding this limitation, our results do reveal the diverse independent contributions of MDD membership to the four cognitive domains, supporting a good differentiation between these functions. An additional limitation of this study may be the statistical methods we used for causal inference. As stated above, the cross-sectional design and correlational nature of the data made it difficult to make accurate causal inferences. However, the dual evidence for the major deficits derived from two independent regression models enhanced the reliability of our inferences. Future studies with longitudinal data, especially data comparing cognitive function in acute depression and remission, will help test our inferences.

## Conclusion

Through a thorough investigation of cognitive function and related risk factors in a large sample of MDD patients and HCs, we found extensive cognitive dysfunction in MDD, involving executive function, processing speed, attention, and memory. In these cognitive domains, executive function and processing speed may serve as two major deficits of MDD arising from different pathways of disease-related detrimental effect on cognition. Specifically, executive function deficit is likely originated from the cumulative deleterious effects of previous depressive episodes on the prefrontal cortex, while the processing speed deficit is possibly derived from temporary depression-severity-related psychomotor retardation. Notwithstanding the influence of various depression-related variables on cognitive function, inter-relationships among different cognitive domains, account for a large proportion of variance in all four cognitive domains, calling for careful considerations in assessing cognitive function in MDD.

## Data Availability Statement

All datasets generated for this study are included in the article/**Supplementary Material**.

## Ethics Statement

The studies involving human participants were reviewed and approved by the Medical Ethics Committees of the Second Xiangya Hospital of Central South University and the Zhumadian Psychiatric Hospital. The patients/participants provided their written informed consent to participate in this study.

## Author Contributions

JL: collected data, conducted statistical analysis, drafted the manuscript, edited, and submitted the manuscript. QD, XL, JS, LZ, MW, YJ, PW, HG, FZ, DY, HL, HF, WG, ML: collected data, reviewed, and revised the manuscript. XZ: edited and revised the manuscript. YZ: conceptualized and designed the study. BL: conceptualized and designed, statistical analysis, critically reviewed, edited, and revised the manuscript. LL: conceptualized and designed, funding the study, collected data, critically reviewed, and revised the manuscript. All authors have approved the final version of this manuscript.

## Funding

This study was supported by the National Science and Technologic Program of China (2015BAI13B02), the Defense Innovative Special Region Program (17-163-17-XZ-004-005-01), and the National Natural Science Foundation of China (81171286, 91232714, and 81601180).

## Conflict of Interest

The authors declare that the research was conducted in the absence of any commercial or financial relationships that could be construed as a potential conflict of interest.
