# Supplementary material for: Exploration of Major Cognitive Deficits in Medication-Free Patients With Major Depressive Disorder
Source: Front Psychiatry. 2019 Nov 12;10:836. doi: 10.3389/fpsyt.2019.00836 (PMC6863061; doi:10.3389/fpsyt.2019.00836)
Supplement: Supplementary file 1 [file Table_1.docx]

**Exploration of major cognitive deficits in medication-free patients with major depressive disorder**

**Supplemental Information**

**Contents**

Table S1. Results of hierarchical regression analyses examining the mediating effect of age and age of onset on the relationships between the alternative variable and the major cognitive deficits. Bold values indicate statistical significance.

Table S1. Results of hierarchical regression analyses examining the mediating effect of age and age of onset on the relationships between the alternative variable and the major cognitive deficits.

| Independent variables  (step 1) | Independent variables  (step 2) | Dependent variable | R^2^ change | F for R^2^ change | P |
| --- | --- | --- | --- | --- | --- |
| Age | Age of onset | Executive  function | .019 | 3.70 | .056 |
|  |  | Processing  speed | .003 | .58 | .446 |
| Age of onset | Age | Executive  function | .092 | 18.16 | **<.001** |
|  |  | Processing  speed | .004 | .72 | .397 |

^Bold values indicate statistical significance.^
